# Facts Do Not Organise Themselves: Whewell and the Conceptual Crisis in Medicine

**DOI:** 10.1111/jep.70617

**Published:** 2026-09-25

**Authors:** Robin J. Evans

**Affiliations:** ^1^ Division of Plastic Surgery University of Tennessee Health Science Centre; Le Bonheur Children's Hospital Memphis Tennessee USA

**Keywords:** consilience, epistemology, evidence‐based medicine, philosophy of medicine, precision medicine, William Whewell

## Abstract

**Rationale, Aims and Objectives:**

Medicine has never possessed more data, yet the conviction that understanding has deepened with it is difficult to sustain. This paper argues that the shortfall is conceptual rather than empirical and turns to the philosophy of science of William Whewell to name it. Evidence‐based medicine tells clinicians how to weigh evidence once it is in hand. The paper aims to show that Whewell describes the earlier and harder task, the forming of the conceptions that make observations count as evidence in the first place.

**Method:**

The analysis is conceptual and historical. Whewell held that facts do not organise themselves but require colligation, the superinduction of a clarifying conception upon observations, and that discovery is most strongly warranted by consilience, the convergence of independent classes of fact upon a single cause. His account is read alongside Peirce on abduction and Fleck on how a scientific fact is held in place, illustrated through a case history of craniosynostosis from Virchow's law to the FGFR discoveries of the 1990s and applied to precision medicine, subspecialisation and artificial intelligence.

**Results:**

In craniosynostosis, children moved between diagnostic categories and a new syndrome came into being without a single new observation, because a conception arrived that reorganised what existing observations were observations of. The contemporary difficulties are versions of the same problem. Precision medicine has delivered where a true cause was secured and stalled where it offered only correlation. Subspecialisation fragments enquiry so that consiliences crossing organ systems go unrecognised. Artificial intelligence extends the power of observation without extending the power of conception.

**Conclusion:**

Medicine has perfected the appraisal of evidence while leaving the formation of concepts largely to chance. The crisis is not one of insufficient data but of insufficient ideas. New conceptions come from prepared minds, and history and philosophy are part of that preparation.

## 1 Introduction

I found them in a bookshop in Boston, on a back shelf between dog‐eared pulp westerns and a stack of church cookbooks: three leather‐bound volumes, fifty dollars each. I was in town for a surgical conference, killing time between sessions, and I nearly walked past them. The author was William Whewell, a carpenter's son from Lancaster who rose to become Master of Trinity College, Cambridge, and who in 1837 published one of the most ambitious intellectual projects of the nineteenth century, a three volume *History of the Inductive Sciences* tracing how each major branch of science had in fact advanced, from antiquity to his own day [1]. Three years later, in the companion *Philosophy of the Inductive Sciences*, he set out an account of scientific reasoning so deliberate that he coined new words for it, among them the word “scientist,” which before Whewell did not exist [2]. I read the set, and over the weeks that followed I came to think that this Victorian had seen something about how knowledge works that medicine has since mislaid.

## 2 Colligation, Consilience and the Prepared Mind

Whewell began from a claim that sounds modest and is not: facts do not organise themselves. The mind must supply the idea that binds them, a process he called colligation, the bringing together of observations by superinducing upon them a conception under which they can be expressed as a general law [2]. His favourite case was Kepler, who made no new measurements of Mars but came to Tycho Brahe's existing tables with the right idea, the ellipse, and so turned a scatter of positions into a law of motion [1]. Kepler added no fact; he added the idea that made the existing facts intelligible. Whewell took this to be the shape of discovery as such: a prepared mind, furnished with clear conceptions, meets the facts and sees what others have looked at without seeing [3].

He pressed the point further. When a conception framed to explain one class of facts turns out to explain a second class it was never devised for, that unlooked‐for convergence is the strongest evidence available that the cause it names is real. Whewell called this consilience, a jumping together of inductions [4]. Newton's theory of gravitation accounted for the orbits of the planets, then for the tides, then for the motion of comets, each established independently and all resolving onto the same law; that convergence was how one could know one had found a *vera causa*, a true cause rather than a serviceable correlation [3].

The idea did not stop with Whewell. Charles Sanders Peirce, who read him closely and thought well of him, was working at the same difficulty half a century later when he argued that neither deduction nor induction accounts for the moment at which a hypothesis first appears. Deduction shows that something must follow and induction that something holds across the cases examined, and neither explains the arrival of the conception under which those cases were gathered in the first place. Peirce gave the missing operation a name of its own, abduction, the inference from a surprising fact to the hypothesis that would render it unsurprising, and when he came to describe how it proceeds he reached for Whewell's word, writing that abduction begins always with the colligation of a variety of separately observed facts [5]. The two classified the operation differently, Whewell placing it within induction and Peirce setting it firmly outside, but they were pointing at the same thing, a creative step at the origin of enquiry that no mechanical rule will produce. It is worth noticing that a question now pressed upon medicine by machine learning, whether a system that finds patterns is doing what a discoverer does, was already the subject of a disagreement between two logicians of the nineteenth century.

Medicine furnishes its own instances, easy to recognise once the pattern has a name. When John Snow plotted the cholera deaths around the Broad Street pump, the marks on his map did not declare their meaning; what made them legible was a conception then far from orthodox, that the disease moved through water rather than through tainted air, and it was that idea, laid over the figures, that turned a cluster of deaths into evidence of a mechanism [6]. The stomach later taught a sharper version of the lesson. Curved bacilli had been seen in gastric tissue for decades and waved away as contaminants, since the reigning conception held that nothing could live in an acid stomach and that ulcers were the work of stress and acid. Warren and Marshall supplied the missing idea, that an organism now called *Helicobacter pylori* colonised the mucosa and provoked inflammation, and at once the separate findings of histology, microbiology and treatment response began to converge [7]. Over the following two decades peptic ulcer disease passed from a chronic complaint suppressed with acid blockade to one often cured outright with antibiotics. The observations had been in hand the whole time. What had been missing was the conception that made them cohere.

## 3 A Problem of Concepts, Not Data

I operate on children born with craniofacial anomalies, patients no single specialty owns, so that my work is as much a matter of crossing institutional boundaries as of crossing tissue planes. I raise it because medicine's predicament is also mine. We are drowning in correlation. The sequencing of the human genome, the growth of multiomics, the spread of electronic health records, national registries, wearable sensors and imaging archives have produced a volume of clinical data without precedent. The abundance is real; it should not be mistaken for progress. The frameworks needed to bind those facts into coherent and usable knowledge have not kept pace, and Ioannidis showed some years ago how unreliable much of the published record had become [8]. The deeper trouble is the one Whewell would have named at once: we have mistaken the accumulation of observations for the advancement of knowledge.

## 4 Craniosynostosis: The Making of a Fact

Whewell describes discovery from the side of the discoverer; there is a companion account, written a century later by a physician, that describes it from the side of the discipline. Ludwik Fleck, a Polish microbiologist, set out in 1935 to explain how the concept of syphilis had come to be what it was, and concluded that it had not so much been discovered as assembled, out of a moral and astrological conception of a carnal affliction, a therapeutic response to mercury, a serological reaction and at last an organism, each stage recasting what the one before it had taken the disease to be Fleck [9]. From this, he drew a distinction that Whewell leaves implicit. Any system of concepts, Fleck argued, holds together relations of two kinds: passive relations, which present themselves as the constraint of the world and are experienced as simply given, and active relations, which are supplied by the thought collective and its style of thought, by training, by institutional arrangement, and by what a community has been taught to regard as worth looking at. The two are not separable in practice, and what a discipline treats as an obvious empirical fact is very often an active relation that has been in place long enough to stop being visible as a choice.

My own field offers a shorter version of the same story, and it is worth telling with some care, because in it the mixture is on plain view. Misshapen skulls had been described since antiquity and catalogued in a Greek vocabulary of appearances, scaphocephaly, trigonocephaly, plagiocephaly, a nomenclature that sorted without explaining. Otto named the condition craniosynostosis in 1830. Two decades later Virchow supplied the conception that turned the names into a subject. Growth, he proposed, is arrested in the plane perpendicular to a prematurely fused suture and continues, compensating, in the plane parallel to it, so that the shape of a child's head follows from which suture has closed and when [10]. This is colligation in the strict sense. Nothing new had been observed; a single generative principle was superinduced upon a set of appearances, and the appearances at once became consequences of a mechanism rather than a catalogue of curiosities. The conception was also, and this matters for what followed, immediately operative. If the suture is the seat of the disorder then the suture is what the surgeon should address, and the earliest operations for the condition were designed directly upon that premise.

Virchow's law then did something that a purely passive relation would not have done. It persisted. Moss argued in 1959 that premature fusion is better read as a consequence than as a cause, the primary disturbance lying in the cranial base and transmitted through the dura, and Delashaw and colleagues later showed that Virchow's rule does not in fact predict the compensatory patterns that produce the recognised deformities [11, 12]. The law is nonetheless still taught as the organising fact of the field. Its durability owes something to its elegance and something to the standing of the man who framed it, and a good deal to how well it suited a discipline that needed a lesion it could excise. That is Fleck's point exactly. A relation we experience as the plain constraint of anatomy has been held in place, in part, by the shape of the specialty that grew up around it.

The sharpest turn came later, from outside the clinic altogether. Through the twentieth century the syndromic forms accumulated as eponyms, each named for the physician who had first assembled a recognisable clinical picture: Apert in 1906, Crouzon in 1912, then Pfeiffer, Jackson and Weiss, Saethre and Chotzen, with more than sixty entities eventually described. They were distinguished by gestalt, by the trained eye of the dysmorphologist, and their boundaries were argued over for decades. Then, across the middle years of the 1990s, mutations in the fibroblast growth factor receptors were identified in one syndrome after another and the arrangement gave way. Apert, Crouzon, Pfeiffer and Jackson Weiss proved to share common ground in *FGFR2*. More striking still, one and the same proline to arginine substitution, falling at the corresponding position in three different receptors, was found to underlie Pfeiffer syndrome in *FGFR1*, Apert syndrome in *FGFR2* and, in *FGFR3*, a population of children who had until then been filed under nonsyndromic coronal synostosis [13]. Muenke and colleagues assembled 61 such individuals from 20 families and described a syndrome that had not previously existed, acknowledging that it could not always be told from its neighbours on clinical grounds [14], and a parallel series found the same mutation in nearly a third of children carrying no syndromic diagnosis at all [15].

Not a single child was examined again, and no new photograph, measurement or radiograph was taken. Children moved between categories, and a category came into being to receive them, because a conception had arrived that reorganised what the existing observations were observations of. Here the accumulation of data and the advancement of understanding come plainly apart. A registry of a hundred thousand affected children, assembled under the eponymous scheme and coded with perfect fidelity to it, could have grown indefinitely without ever yielding the regrouping, because the scheme that generated the records had already settled what could be recorded. Whewell would have called the new arrangement a colligation, and would have found in the recurrence of a single substitution across three receptors and three formerly separate syndromes precisely the jumping together of inductions that distinguishes a true cause from a serviceable classification.

One further episode completes the pattern, and it runs the other way. In 1992, the American Academy of Paediatrics advised that healthy infants be laid on their backs to sleep, a recommendation that has saved a great many lives and had nothing whatever to do with skull surgery. Within a few years craniofacial centres were seeing an unprecedented number of infants with flattened and asymmetric occiputs, a deformation produced by external moulding of an intrinsically normal cranium [16, 17]. The conceptual scheme in hand had a category ready to receive them, posterior synostotic plagiocephaly, and a substantial number of these children were absorbed into it and taken to theatre for cranial vault expansion before the two conditions were properly distinguished [18]. A social decision altered the population of skulls arriving in clinic, the volume of observation rose steeply, and diagnostic accuracy fell, because the governing conception had been formed for a population that no longer described the one in front of us. Fleck would have recognised the active relation, and so, I suspect, would anyone who was in practice at the time.

## 5 Evidence‐Based Medicine and the Formation of Concepts

This complaint is not new to philosophers of medicine, and the argument here is meant to enter their conversation rather than to start one. For three decades evidence‐based medicine has organised that conversation around a single question: what ought to count as evidence, and how should it bear on the care of the patient in front of us. Its achievement was to impose discipline on clinical observation, ranking study designs by their power to exclude bias and placing the randomised trial and the systematic review at the summit of that ranking. The criticism it has drawn concerns not the discipline but its sufficiency. Cartwright has argued that a trial, however rigorous, shows that an intervention worked in the population studied, and that the step from there to whether it will work in this patient runs along a long road laid with causal assumptions the trial does not itself provide [19]. Howick, writing from within the movement, has nonetheless argued that mechanistic reasoning and clinical expertise belong inside the evidence base rather than beneath it [20].

The same misgiving has been pressed from the side of the clinician as a knower. van Baalen and Boon hold that the limits of population level evidence call not for more data but for what they name epistemological responsibility: the clinician must combine unlike kinds of knowledge into a judgement about the particular case, an act no hierarchy of evidence can carry out on her behalf [21]. Chin‐Yee and Upshur, drawing on Collingwood, describe clinical understanding as nearer to historical reasoning than to the filing of a case under a general law, a mode of thought that reconstructs the particular rather than merely classifying it [22]. What these arguments share is the sense that evidence‐based medicine, for all its rigour, accounts for only half of medical knowing. At bottom it is an epistemology of appraisal: it governs how observations are weighed once they have been gathered and once the question they answer has been framed, but it is silent on where that framing originates.

That is the half Whewell describes. Colligation is the act by which a question is posed and a set of facts is constituted as evidence for something at all. Brahe's measurements were not yet evidence for an elliptical orbit until Kepler conceived the ellipse; the deaths around the Broad Street pump were not yet evidence of waterborne contagion until Snow supplied the idea that made them so. Evidence‐based medicine takes up the work at the point where the governing conception is already in hand and the hypotheses are already on the table awaiting test. Whewell's interest lies in the earlier and harder moment, the forming of the conception itself, which he regarded as the creative and most demanding part of enquiry, and which no quantity of well‐appraised observation will yield on its own. A medicine that has perfected the appraisal of evidence while neglecting the formation of concepts has equipped itself admirably for the second half of enquiry while leaving the first largely to chance.

## 6 Precision Medicine: Brahe Without Kepler

The cost of neglecting concept‐formation is easiest to see in precision medicine. Its promise was definite: genomic, proteomic and phenotypic data converging to individualise diagnosis and treatment, and the infrastructure built to pursue that promise is real and considerable. Two decades on, the returns have been uneven, in a pattern Whewell's distinction helps explain. Where precision medicine has transformed care, it has done so on the strength of a conception and not merely a measurement. Chronic myeloid leukaemia is the canonical case: the disease was traced to a single chromosomal translocation that produces a constitutively active fusion kinase, and once that causal idea was secure a drug built to block the kinase, imatinib, turned a lethal leukaemia into a condition many patients live with for decades. This is Kepler rather than Brahe: the measurements counted because an idea told us what they meant.

The contrast is the yield of large‐scale association. Genome‐wide association studies have tied thousands of common variants to common diseases, yet for most such traits the variants identified account for only a part of the heritability that family studies imply, a shortfall conspicuous enough to have earned its own name, the problem of missing heritability [23]. These studies are feats of measurement and of statistical care. What they have mostly not delivered is the unifying conception that would explain why the variants matter and how they act; they have handed us Brahe's catalogue without Kepler's law. Basket trials that match drugs to mutations across tumour types have taught a kindred lesson, their response rates modest once one steps outside the few targets whose biology is well understood. Precision medicine has delivered where a true cause was found, and stalled where it had only correlation to offer.

The same underdevelopment surfaces in the words clinicians use with most confidence. We speak every day of inflammation, of frailty, of resilience, and these remain loosely drawn conceptions, nearer to placeholders than to the exact ideas Whewell thought the precondition of discovery [24]. Precision of measurement has outrun precision of conception, and it is the latter that performs the explanatory work.

## 7 Subspecialisation and the Invisibility of Consilience

If precision medicine shows conceptions failing to form, subspecialisation shows the ones we already have failing to meet. A cardiologist, an endocrinologist, a psychiatrist and a geneticist may each be studying a different face of one underlying process, whether metabolic syndrome, systemic inflammation or the gut‐brain axis, without any of them recognising that their separate findings are converging. No one stands far enough back to watch the inductions jump together. The institutional design of modern medicine, arranged around organ systems and departmental lines, works against the recognition of causes that cross those lines. We have been here before. The germ theory of disease was itself a consilience: miasma was a vague and poorly drawn conception, and when it gave way to the precise idea of specific microbial pathogens, observations from surgery, obstetrics, public health and epidemiology were eventually drawn together under a single account [25]. How many comparable convergences surround us now, unseen because our structures are not built to see them?

I watch a version of this in my own work, where imaging data, developmental assessments and morphometric measurements drawn from three disciplines have begun to converge on a common mechanism in children with cranial vault anomalies. The convergence became visible only because the people involved were willing to cross departmental boundaries that were never meant to be crossed. Galison's name for such a place is a trading zone, a site at which communities holding incompatible vocabularies work out a local means of exchange without first agreeing on fundamentals [26]. Whewell would have called it a consilience in progress. From within the work it looks like nothing so grand, only the ordinary business of caring for children who fall between the specialties.

## 8 Artificial Intelligence: Pattern Without Conception

Artificial intelligence presses the distinction to its limit. A model can find statistical regularities across millions of data points at a speed and scale no clinician could approach, and that is a real extension of our powers of observation. It is not, in Whewell's sense, an extension of our powers of conception. Pattern recognition is not colligation. A network can report that certain imaging features predict an outcome while offering no idea of why they do, and a prediction without a conception is Brahe's catalogue produced at machine speed. Verghese and colleagues have argued that what the computer needs is a physician, and that humanism and machine learning are complements rather than rivals [27]; Shah and colleagues have traced how rapidly large language models have entered clinical work and the questions they raise for clinical reasoning [28].

Shaw raises a kindred difficulty about the data themselves. A genuine scientific instrument, on his account, is one built to a conceptual specification stable enough to be shared across a community, so that what it records can be interpreted by anyone who understands its design; most large data systems have instead accreted out of heterogeneous and shifting purposes, and the traces they hold are better approached as historical artefacts calling for interpretation than as measurements awaiting aggregation [29]. The electronic health record is the obvious instance, built for billing and for medicolegal defence and answering clinical questions only insofar as we contrive to forget what it was built to do. Recht has recently traced the ancestry of the assumption lying underneath all of this, following the notion that every decision is at bottom a statistical question of risk from wartime optimisation and game theory through the randomised trial and on into machine learning, and arguing that a conception of rationality serviceable within its range has been asked to settle questions it never could [30]. His history is a useful corrective to the habit of treating evidence‐based medicine and artificial intelligence as two separate difficulties, since both inherit the same account of what a decision is.

Whewell would have granted the power and located its limit. Induction, he insisted, adds a ‘new element’ to the instances ‘by the very act of thought by which they were combined’ [2], and that act is not computation. These systems can give medicine the most precise observations it has ever held. They cannot supply the ellipse.

## 9 Implications: How Prepared Minds Get Prepared

The diagnosis has a practical consequence. If discovery depends on minds prepared with conceptions drawn from more than one domain, then the relentless drive towards ever narrower specialisation is, in part, a drive against discovery. The physician who reads only within her own field is a mind stocked with a single conception, able to colligate within her territory but blind to the consiliences that cross it. Whewell finished his *History* before he wrote his *Philosophy* because he held that one cannot theorise about how science works without first studying how it has actually worked [31]. The medical humanities, so often treated as graceful additions to a scientific training, turn out to be closer to working equipment: they are part of how a mind is furnished to colligate at all, and a curriculum that treats them as ornament strips out the very faculty it most needs to cultivate [32].

Fleck gives that claim a firmer footing than well roundedness can. If the conceptions a clinician works with are held in place partly by active relations, by training and institutional habit and the style of thought of the collective to which she belongs, then the disciplines that make those relations visible, history, philosophy, and the study of how a community comes to find one description obvious and another unthinkable, are not adornments upon a scientific education. They are the means by which a practitioner can tell which of her certainties are constraints of the world and which are inheritances of her guild. A physician who cannot draw that distinction is not in a position to form a new conception, because she cannot see the one she already holds.

## 10 Conclusion

Whewell died in 1866, thrown from his horse outside Cambridge, and asked at the last that the curtains be drawn back so that he might see the great court of Trinity once more [33]. Philosophers of science have rediscovered his work with growing admiration; medicine has barely touched it. That is a loss worth repairing. We live at a moment when medicine generates facts faster than it can make sense of them, when artificial intelligence offers pattern in the absence of meaning, and when the silos of subspecialty obscure the very convergences that would signal real understanding. Whewell's framework, colligation, consilience and the prepared mind, offers medicine a diagnosis and, in the same gesture, a direction. The question is not whether we have enough data. It is whether we have the right ideas. The answer, on Whewell's account, lies not in the data but in the conceptions we bring to it, and some of those may be waiting on a shelf to be recognised, much like three leather‐bound volumes priced at fifty dollars each.

## Funding

The author has nothing to report.

## Ethics Statement

This article is a conceptual and philosophical analysis. It did not involve research on human participants or animals, and no identifiable patient data are reported; ethics committee approval was therefore not required.

## Conflicts of Interest

Dr. Evans had a consulting relationship with Mectron Piezosurgery, outside the submitted work, which has since concluded. No other disclosures were reported.

## Data Availability

Data sharing is not applicable to this article as no new data were created or analysed in this study.
